# Key actors in cancer therapy: epigenetic modifiers

**DOI:** 10.3906/biy-1903-39

**Published:** 2019-06-13

**Authors:** Remzi Okan AKAR, Selin SELVİ, Engin ULUKAYA, Nazlıhan AZTOPAL

**Affiliations:** 1 Department of Cancer Biology and Pharmacology, Institute of Health Sciences, İstinye University, İstanbul, Turkey; 2 Department of Medical Biochemistry, Faculty of Medicine, İstinye University, İstanbul, Turkey; 3 Department of Molecular Biology and Genetics, Faculty of Science and Literature, İstinye University, İstanbul, Turkey

**Keywords:** Epigenetic modifications, RNA epigenetic, anticancer drugs, cancer stem cells

## Abstract

Epigenetic reprogramming plays a crucial role in the tumorigenicity and maintenance of tumor-specific gene expression that especially occurs through DNA methylation and/or histone modifications. It has well-defined mechanisms. It is known that alterations in the DNA methylation pattern and/or the loss of specific histone acetylation/methylation markers are related to several hallmarks of cancer, such as drug resistance, stemness, epithelial-mesenchymal transition, and metastasis. It has also recently been highlighted that epigenetic alterations are critical for the regulation of the stemlike properties of cancer cells (tumor-initiating cells; cancer stem cells). Cancer stem cells are thought to be responsible for the recurrence of cancer which makes the patient return to the clinic with metastatic tumor tissue. Hence, the dysregulation of epigenetic machinery represents potential new therapeutic targets. Therefore, compounds with epigenetic activities have become crucial for developing new therapy regimens (e.g., antimetastatic agents) in the fight against cancer. Here, we review the epigenetic modifiers that have already been used in the clinic and/or in clinical trials, related preclinical studies in cancer therapy, and the smart combination strategies that target cancer stem cells along with the other cancer cells. The emerging role of epitranscriptome (RNA epigenetic) in cancer therapy has also been included in this review as a new avenue and potential target for the better management of cancer-beneficial epigenetic machinery.

## 1. Introduction

Success in cancer treatment is still not satisfactory, although new chemotherapeutics have recently been introduced in the clinic. For a decade, it has been considered that the most important reason for the poor success rate in cancer therapy is the presence of cancer stem cells (CSCs) that represent tumor heterogenicity. It has been shown that CSCs are resistant to treatment and that they play a crucial role in recurrence (Easwaran et al., 2014; Toh et al., 2017). It is now known that classic genetics alone is insufficient to explain the diversity of phenotypes within a population. Therefore, new therapy regimes which include epigenetic modifiers have begun assessment in clinical trials (Samavat et al., 2016; Azad et al., 2017; Von Hoff et al., 2018). Recent studies suggest differential alterations in epigenetic mechanisms for several cancers such as breast, colon, lung, prostate, and brain tumors (glioblastoma multiforme) (Bachmann et al., 2006; Kagara et al., 2012; Vedeld et al., 2014). It has been reported that gene-specific hypermethylation of DNA and/or modulation of the histone acetylation program are the most abundant modifications of tumorigenesis that can be reversed by the epigenetic modifiers (Fraga et al., 2005). Furthermore, it has been shown that use of these modulators in combination with conventional chemotherapeutics exhibits chemo-prevention activity against different types of cancer (Fang et al., 2010; Matei et al., 2012; Von Hoff et al., 2018). There are also several preclinical studies that have shown promising results in targeting CSCs via these drugs (Tian et al., 2012; Liu et al., 2015; Jiang et al., 2018). In addition, identification of the role of epitranscriptome in cancer-related processes has become a new research area due to its association with aggressive tumor character (Zhang et al., 2017). There are a few studies regarding the modulation of this process resulting in the elimination of cancerous cells as well as CSCs, implying new possible targets in this area (Zhang et al., 2016; Cui et al., 2017; Zhao et al., 2017). Thus, the current review aims to address the following questions: i) How does epigenetic machinery contribute to tumor growth and progression? ii) What are the current clinical and preclinical studies on the usage of epigenetic modifiers in cancer therapy? iii) What are the benefits of using these modifiers to target CSCs? iv) How can combinatorial therapy be effectively used to target/inhibit these cells? v) What is the role of epitranscriptome in cancer therapy?

## 2. Epigenetic mechanisms

The term epigenetics was first used by Conrad Waddington in 1942 and is defined as all changes inherited through the alteration of gene expression without alteration of the DNA sequence (Herceg, 2007). Epigenetic gene regulation occurs through covalent modifications of both DNA and chromatin, and modulates gene transcription by opening the chromatin structure (euchromatin, active gene transcription) or by providing a condensed DNA construct (heterochromatin, suppressed gene expression) (Meissner, 2010). DNA methylation and histone modifications are two important epigenetic modifications included in several biological processes such as embryonic development, cellular memory, cell, and tissue-specific gene expression, differentiation, and adult tissue maintenance (Meissner, 2010; Tammen et al., 2013). On the other hand, noncoding RNAs such as piRNAs, miRNAs, and long noncoding RNAs are also important epigenetic regulators discussed in detail elsewhere, but they are not the focus of this review (Wei et al., 2017).

### 2.1. DNA methylation

DNA methylation refers to the addition of a methyl (CH3) group covalently to the cytosine bases of the CG dinucleotides (CpG) at the fifth carbon position by the DNA methyl transferases (DNMT), which changes cell functions by altering gene expression (Bird, 2002). CpG islands refer to the frequency of CG sequences, which is higher than that of other regions in the genome in which chromatin structure and gene expression are modulated (Baylin, 2005). The promoters of transcriptionally active genes (housekeeping genes and most regulatory genes) are associated with this region and are unmethylated. The methylation of cytosine in CpG islands is associated with transcriptional silencing. It is hence a regulatory mechanism for gene expression (Bird, 2002). Gene silencing by DNA methylation occurs through the suppression of transcription factors binding to the promoter region or induction of binding proteins (MBD; methylated CpG binding proteins) which are specific to these methylated regions (Klose and Bird, 2006). 

### 2.2. Histone modifications

DNA and histones are organized into nucleosomes to form the chromatin structure in eukaryotes. Each nucleosome core consists of a histone octamer containing a pair of H2A, H2B, H3, and H4 histone proteins, and each nucleosome is connected with the linker DNA. The linker H1 histone binds to the entry and exit region of DNA on the histone core (Annunziato, 2008). Each histone core has a long N-terminal amino acid tail that extends from the nucleosome structure, and these histone tails exhibit various posttranslational modifications that control the chromatin structure, such as acetylation, methylation, phosphorylation, ubiquitination. These specific histone modifications on lysine (K), serine (S), and arginine (R) residues are known as histone code and may be an activator or a suppressor according to the specific region modified. Modulation of histones are regulated by histone acetyl transferases (HATs), histone methyltransferases (HMTs), histone deacetylases (HDACs), and histone demethylases (HDMs) through the addition or removal of methyl or acetyl groups to/from the histone tails (Shi, 2007; Haberland et al., 2009). Generally, acetylation provides the euchromatin state through the charge neutralization between DNA and histones, and is associated with transcriptional activity. On the other hand, methylation can activate or repress transcription depending on which residue is modified (Bernstein et al., 2007; Bannister and Kouzarides, 2011). For example, while the addition of 3 methyl groups to the 4th lysine residue of H3 histone protein (H3K4me3) is an active mark, trimethylation of the 27th lysine residue of H3 histone protein (H3K27me3) is a repressive mark (Jenuwein and Allis, 2001). Polycomb group (PcG) proteins are exclusive chromatin-modifying transcriptional repressors that determine the cell’s fate by regulation of pluripotency and/or differentiation of cells. Modulation of histones by PcG proteins provides the maintenance of pluripotency in early embryonic development, and is also responsible for the silencing of pluripotency genes following differentiation (Bernstein et al., 2006). Enhancer of zeste homolog 2 (Ezh2) is a well-known PcG protein and an HMT catalyzing trimethylation of lysine 27 of the H3 mark, and is essential for the maintenance of embryonic stem cell pluripotency (Vire et al., 2006). 

## 3. Epigenetic status in cancer

The cancer genome is generally hypomethylated, which causes increased genomic instability, as well as activation of prosurvival and growth-promoting genes (Jones and Baylin, 2007). On the other hand, it is known that there is gene-specific hypermethylation in tumors resulting in transcriptional inactivation of tumor suppressor genes such as Rb, BRCA1, and MLH1 (Baylin, 2005; Jones and Baylin, 2007). In addition to alterations in DNA, modification of histones is also involved in the process of tumorigenesis. Loss of acetylation at lysine 16 (H4K16ac, active mark) and trimethylation at lysine 20 of histone H4 (H4K20me3, repressive mark**)** have been indicated as common hallmarks of various forms of cancer (Fraga et al., 2005). HDACs are often found overexpressed in various types of cancer and thus have become a major target of epigenetic therapy (Halkidou et al., 2004; Noh et al., 2006; Hayashi et al., 2010). Along with HDACs, deregulation of HMTs is related to tumor initiation and progression, and is especially responsible for methylation at lysines 4, 9, and 27 of histone H3 (Nguyen et al., 2002; Valk-Lingbeek et al., 2004; Kondo et al., 2008). Elevated levels of Ezh2 protein have been reported in prostate and breast cancer (Valk-Lingbeek et al., 2004). Furthermore, recent studies have stated that Ezh2 overexpression is associated with aggressiveness and progression of different cancer types such as prostate, breast, bladder, and endometrial cancer, as well as melanoma (Bachmann et al., 2006; Clermont et al., 2017).Research on epigenetic therapy which has especially focused on DNMT and HDAC inhibitors as enlightened epigenetic mechanisms in cancer accelerated in the early 2000s. It has been shown that cancer-beneficial epigenetic modifications are critical for tumor initiation and progression because of these modifications related to the hallmarks of cancer. Differential epigenetic processing has been stated in normal cells, cancer cells, and CSCs (Figure). Hence, research on epigenetic therapy has gained momentum in recent years to target these mechanisms. Newly discovered inhibitors have been introduced in addition to DNMT and HDAC inhibitors, such as bromodomain and extraterminal protein (BET), histone methyltransferase (HMT), protein arginine methyltransferase (PRMT), and lysine-specific demethylase 1A (LSD1/KDM1A) inhibitors, which are able to target different epigenetic mechanisms (Jones et al., 2016). It has been shown in these studies that epigenetic drugs (epidrugs) have promising results for the treatment of cancer. Currently, there are 6 FDA-approved epigenetic drugs used in oncology, and more than 50 epigenetic drug candidates are being examined in preclinical and clinical trials. Moreover, combinatorial usage of epidrugs is in preclinical and/or clinical trials(Tables 1 and 2).

**Figure:**
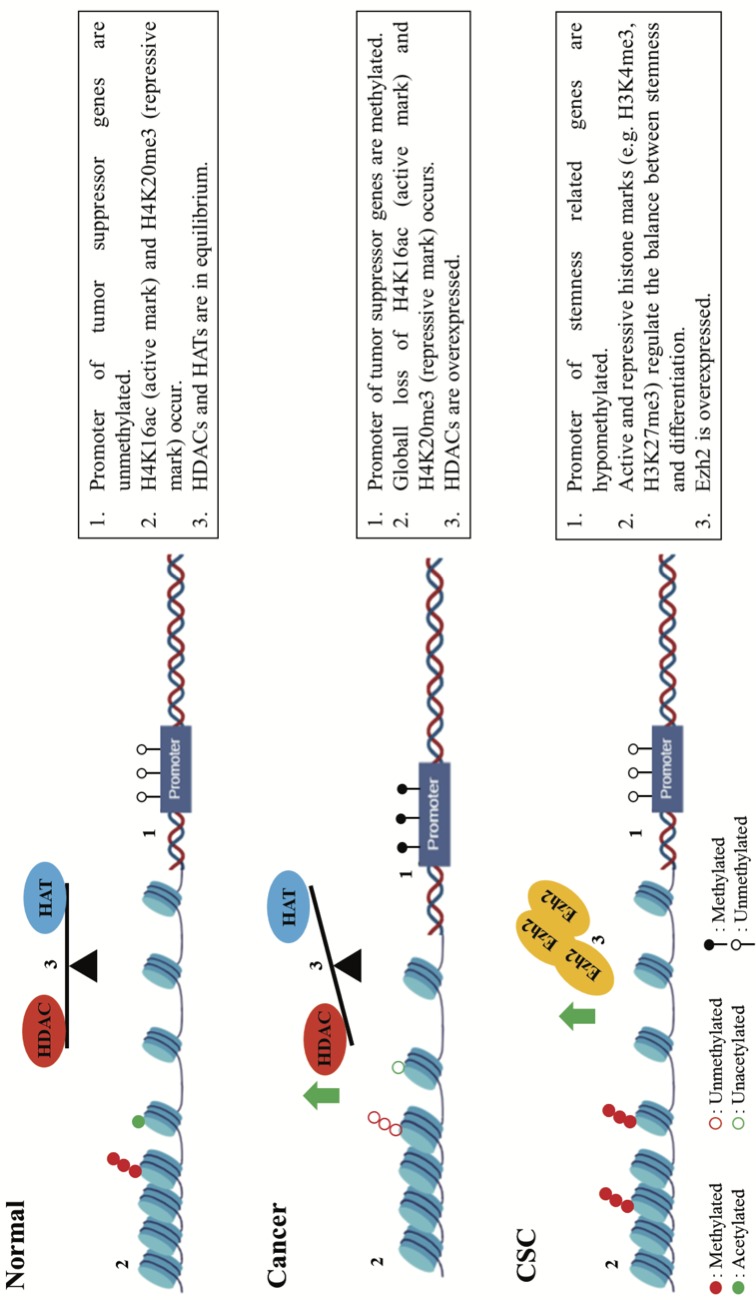
Overview of the hot top epigenetic alterations in normal cells, cancer, and CSCs.

**Table 1:** Epigenetic modifiers used in clinic trials.

Epigenetic drug	Status	Effect/limitation(alone)	Combinatorial therapy	Effect/limitation(combined)	References
Azacytidine	Phase 1Phase 3	Delayed transformation fromMDS to AML Serious side effects. Resulted with partial response andstable disease (CC-486).	+Entinostat (Phase 1/2)+VPA (Phase 1/2)	Increased median PFS and overall survival. Demethylation of tumor associated genes.	Kornblith et al., 2002Braiteh et al., 2008 Juergens et al., 2011Azad et al., 2017Von Hoff et al., 2018
Decitabine	Phase 2 Phase 3	Increased PFS Delayed transformation fromMDS to AML Toxicity: nausea, allergic reactions, neutropenia, fatigue	+Carboplatin (Phase 1)	Increased sensitivity to carboplatin Decrement in global DNA methylation	Kantarjian et al., 2006Fang et al., 2010Matei et al., 2012
EPG	Phase 2	Decrement in circulating tumor cells	N/A	N/A	Samavat et al., 2016
SAHA	Phase 1/2	No objective antitumor activity No complete or partial responsesbutstable disease	+Decitabine by the following chemotherapy: Vincristine, Dexamethasone, Mitoxantrone (Phase 1/2)	Reported encouraging results to reverse chemotherapy resistance in relapsed/refractory ALL	Luu et al., 2008 Modesitt et al., 2008Traynor et al., 2009Burke et al., 2014
Romidepsin	Phase 3	Limited activity alone	+ Erlotinib (Phase 1)	Encouraging findings for disease control	Haigentz et al., 2012Gerber et al. 2015
Belinostat	Phase 2	Complete and durable responseswith manageable toxicity	+ Cisplatin, Doxorubicin, and Cyclophosphamide (Phase 1/2)	Combination is active without additive toxicities	Thomas et al., 2014O’Connor et al., 2015
Panobinostat	Phase 3	Increased PFS	+Bortezomib andDexamethasone (Phase 3)	The overall survival benefit	San-Miguel et al., 2014Richardson et al., 2016 San-Miguel et al., 2016
VPA	Phase 2	Clinically useful in low-risk MDS	+ ATRA and 5-Azacytidine+ Standard radiation therapyand temozolomide	A set of genes is associated with clinical response Encouraging findings for improved outcomes	Kuendgen et al., 2005Raffoux et al., 2010Krauze et al., 2015

**Table 2:** Epigenetic modifiers used in preclinical studies.

Epigeneticdrug	Effect/limitation(alone)	Combinatorialtherapy	Effect/limitation(combined)	References
Azacytidine	Antigrowth activity Immunomodulatory effect	+ Temozolomide+ Doxorubicin	Increased chemosensitivity	Li et al., 2014Khan et al., 2017Gailhouste et al., 2018Yamashita et al. 2018
SGI-110	Immunomodulatory effect	+Oxaliplatin+Cisplatin	Chemosensitizer Overcame cisplatin resistance	Fang et al., 2014Srivastava et al., 2015Kuang et al., 2015
SAHA	Cytotoxic activity Relatively low toxicity against normal cells	+Doxorubicine+Cisplatin+Bortezomib+Tyrosine kinase inhibitors (TKIs)+Olaparib	Increased sensitivity to anticancer agents andapoptosis Overcome EGFR TKI resistance and increasedapoptosis Increased apoptotic and autophagic cell death	Nawrocki et al., 2006Vijayaraghavalu et al., 2012Lee et al., 2015bMin et al., 2015Grabarska et al., 2017
Romidepsin	Antigrowth activity	+Cisplatin +Etoposide	Synergistic effect	Luchenko et al., 2011Li et al., 2016
Belinostat	Inhibition of proliferation and metastasis	+CYC202	Increased apoptosis	Qian et al., 2008Ong et al., 2016
VPA	Induction of differentiation and apoptosis	+Paclitaxel+Cisplatin	Increased sensitivity to paclitaxel Overcame cisplatin resistance	Stockhausen et al., 2005Catalono et al., 2007Cipro et al., 2012Aztopal et al., 2018b
Panobinostat	Decreased proliferation and increased cell death Decreased tumorigenesis	+Gefitinib	Overcame gefitinib resistance	Tate et al., 2012Lee et al., 2017
DZNep	Decrement in global H3K27me3 Inhibition of proliferation and migration	+Nutlin-3+SAHA	Induced apoptosis	Kemp et al., 2012Takashina et al., 2016Zhou et al., 2018
Decitabine	Anticancer activity	+Carboplatin +Cisplatin	Increased sensitivity to carboplatin and cisplatin Increased global DNA hypomethylation	Steele et al., 2009Budden et al., 2016
JQ1	Inhibition of angiogenesis Inhibition of PD-L1 expression	+Rapamycin	Effective chemotherapeutic option forosteosarcoma treatment	Lee et al., 2015aZhu et al., 2016Leal et al. 2017da Motta et al., 2017
EPG	Inhibition of tumor progression and metastasis Reduced circulating tumor cells Decreased stemness	+5-FU	Enhanced chemosensitivity	Kim et al., 2014Samavat et al., 2016Toden et al., 2016Chen et al., 2017Jiang et al., 2018
Curcumin	Decreased CpG methylation of DLEC1 Inhibited anchorage-independent growth	+Folfox	Enhanced chemotherapy induced apoptosis	Guo et al., 2015James et al., 2015

### 3.1. Targeting DNA modifications in cancer therapy

Among the most frequently studied DNA modifiers are DNMT inhibitors that are modified forms of cytosine and act as cytosine analogs. Currently, two of them, azacytidine and decitabine, are approved by the FDA for clinical usage (Verma and Kumar, 2018). Their mechanism of action is that decitabine incorporates into DNA whereas azacytidine incorporates into both RNA and DNA; both then lead to degradation of DNA methyltransferases (Toh et al., 2017). They were first developed as cytotoxic agents against cancer in 1964. Subsequent studies have shown that low-dose azacytidine or decitabine inhibits DNA methylation (Christman, 2002). In 2004 and 2006 respectively, the FDA approved azacytidine and decitabine following reassuring results on the treatment of myelodysplastic syndrome (MDS), which is considered preleukemia (Sato et al., 2017). Furthermore, both azacytidine and decitabine have shown promising results on AML and CML because they delay the time to AML transformation from MDS or death (Kornblith et al., 2002; Kantarjian et al., 2006). There have been 13 completed Phase 3 clinical trials on azacytidine treatment in leukemia and MDS. However, because of the serious side effects observed in these studies, new interventions are being tried in 9 active trials. There are over 200 clinical trials in different phases on different type of cancers with azacytidine and decitabine. Matei et al. 2012 showed in a Phase 2 study that low-dose decitabine affects the gene methylation of cancer-related pathways, restores sensitivity to carboplatin, and increases response rate and progression-free survival (PFS) in ovarian cancer patients (Matei et al., 2012). Another phase study reported that a low-dose decitabine and carboplatin combination is well tolerated, and resulted in DNA hypomethylation in patients with ovarian cancer (Fang et al., 2010). Phase 1/2 studies using combinatorial therapy with azacytidine and valproic acid or entinostat showed that tumor-associated methylated genes were demethylated. However, some side effects occurred, such as neutropenic fever, thrombocytopenia, and anemia (Braiteh et al., 2008; Juergens et al., 2011). Sequential administration of azacytidine and lenalidomide, but not in high doses, has shown clinical activity in patients 60 years or older with AML (Medeiros et al., 2018). CC-486, an oral formulation of azacytidine, has resulted in partial response and stable disease in nasopharyngeal cancer patients. The same Phase 1 study showed that CC-486 alone or in combination with carboplatin or nab-paclitaxel (nanoparticle albumin-bound) are well tolerated and no drug-drug interactions were detected (Von Hoff et al., 2018). The immunomodulatory effect of azacytidine was identified in breast, colon, and ovarian cancer cells in vitro with alteration of immune gene methylation (Li et al., 2014). This data suggests that usage of azacytidine in combination with immunotherapy may be an option for cancer treatment. Small molecule inhibitors have been developed that inhibit DNA methylation without being incorporated into DNA (Brueckner et al., 2005). SGI-110 (guadecitabine), the prodrug form of decitabine, is a small molecule of DNA methylation inhibitor. SGI-110 has been used to increase chemosensitivity in colon cancer and ovarian cancer with combinatorial therapies in vitro and in vivo (Fang et al., 2014). There are clinical trials on SGI-110 in Phases 2 and 3 on different types of solid cancers, but results have not been published yet. On the other hand, SGI-110 has immunomodulatory activity similar to that of azacytidine (Li et al., 2014; Srivastava et al., 2015). Hence, combination therapy of DNA modifiers with immunotherapy may produce promising results for cancer treatment.Additionally, there are several natural compounds that affect DNMTs. One of these compounds is epigallocatechin gallate (EPG), which has exhibited promising results in Phases 2 and 3 clinical trials completed on skin, prostate, breast, and colon cancers. One of the studies showed that EPG containing green tea extract significantly reduced circulating tumor cells in breast cancer patients (Samavat et al., 2016). Kim et al. 2014 showed that EPG inhibits progression of prostate cancer and metastasis in TRAMP mice (Kim et al., 2014). Another natural compound that affects DNA methylation is curcumin (Liu et al., 2009). It has one clinical trial that is recruiting for prostate cancer in Phase 3, as well as ongoing studies in Phase 2 against various type of cancers such as breast, pancreatic, endometrial, prostate, and colorectal cancers. It has been shown in vitro that curcumin reduces CpG methylation of tumor suppressor DLEC1 gene promoter and inhibits anchorage-independent growth in HT29 human colorectal cancer cells (Guo et al., 2015). There are several challenges for the usage of epigenetic drugs alone in the treatment of solid tumors. One of the limitations for DNMT inhibitors is the ability to provide the penetration of the drug into tumor tissue and targeting cancer cells. Furthermore, epidrugs affect multiple pathways because they are not selective; they target global epigenetic modifications. Another limitation is that they work on actively dividing cells (Issa and Kantarjian, 2009). Therefore, there are plenty of combinatorial therapies with DNMT inhibitors and other epigenetic drugs, conventional chemotherapy, or inhibitors. Azacytidine in combination with entinostat, a class I HDAC inhibitor, has been studied in treating 46 patients from 2 cohorts with metastatic colorectal cancer in a Phase 2 trial. The median PFS was stated as 1.9 months and overall survival was stated as 5.6 months in cohort 1 and 8.3 months in cohort 2 (Azad et al., 2017). Decitabine and carboplatin have been tested in platinum-resistant ovarian cancer patients; it was found that low-dose decitabine affects cancer-related DNA methylation and restores sensitivity to carboplatin (Fang et al., 2010; Matei et al., 2012).

### 3.2. Targeting histone modifications in cancer therapy

It is known that HDACs are overexpressed in various cancer types (Halkidou et al., 2004; Zhu et al., 2004; Zhang et al., 2005; Noh et al., 2006). Therefore, targeting HDACs has been considered an important strategy in cancer treatment. The 5 currently FDA-approved HDAC inhibitors are vorinostat (SAHA) for cutaneous T-cell lymphoma, romidepsin for cutaneous/peripheral T-cell lymphoma, belinostat for peripheral T-cell lymphoma, panobinostat for multiple myeloma, and valproic acid (VPA) for epilepsy, bipolar disorder, and migraine (Eckschlager et al., 2017; Verma and Kumar, 2018). There have been 5 completed Phase 3 clinical trials with SAHA on lung cancer, glioma, multiple myeloma, and leukemia, but results have not been posted yet. It has been reported that SAHA did not exhibit antitumor activity against head and neck cancers, breast, ovarian, colorectal, and nonsmall cell lung cancers in Phase 2 clinical trials (Blumenschein et al., 2008; Luu et al., 2008; Modesitt et al., 2008; Vansteenkiste et al., 2008; Traynor et al., 2009). On the other hand, combinatorial therapies with SAHA have promising results in preclinical studies. Combinatorial therapy with SAHA and olaparib, a PARP inhibitor synergistically inhibited growth of triple negative breast cancer cells, caused DNA damage and increased apoptotic and autophagic cell death in vitro and in vivo (Min et al., 2015). Another study showed that a combination of SAHA and tyrosine kinase inhibitors (afatinib and WZ4002, third generation of TKIs) overcame EGFR TKI resistance in lung cancer cells and decreased cell viability both in vivo and in vitro (Lee et al., 2015b). It has also been reported that pretreatment with SAHA increases the cytotoxicity of chemotherapy, even in chemo-resistant cells (Kim et al., 2003). SAHA in combination with bortezomib, a proteosome inhibitor, exhibited a synergistic and potential effect in multiple myeloma, pancreatic cancer, and nude mice xenografts (Nawrocki et al., 2006). Vijayaraghavalu et al. 2012 demonstrated that treatment with decitabine or SAHA sensitized chemoresistant MCF-7 breast cancer cells to doxorubicin treatment. It has been shown that belinostat inhibited growth of prostate cancer cells in vitro, and reduced metastasis in vivo**(Qian et al., 2008). Another study showed that belinostat and a CDK inhibitor (CYC202) significantly reduced proliferation and increased apoptosis via upregulating p53 and BID activation (Ong et al., 2016).Romidepsin, a cyclicpeptide HDAC inhibitor and natural product isolated from a bacterium, has been tested in a completed Phase 3 trial in 27 countries, including Turkey (Falkenberg and Johnstone, 2014). Results indicate that only 17% of patients showed a complete response and 17% of patients showed a partial response to romidepsin (O’Connor et al., 2015). There is an active study with romidepsin in Phase 3 against peripheral T-cell lymphoma. The aim of the study is to compare the efficacy of romidepsin when administered with CHOP (cyclophosphamide, doxorubicin, vincristine, and prednisone) versus CHOP alone in patients previously untreated for peripheral T-cell lymphoma. The effect of romidepsin on solid tumors has been studied in 21 completed Phase 2 trials. One of these studies was on radioactive iodine used on irresponsive recurrent and metastatic thyroid cancer; it was reported that “no RECIST (Response Evaluation Criteria in Solid Tumors) major responses have been seen” (Sherman et al., 2013). In another study, no objective response to romidepsin was observed in patients with recurrent/metastatic head and neck cancer, as romidepsin alone has limited activity in treating squamous cell carcinoma of the head and neck (Haigentz Jr et al., 2012). Therefore, combinatorial therapies are considered important. It has been reported that the combination of romidepsin and cisplatin or etoposide exhibits a synergistic effect on lung cancer cells in vitro (Luchenko et al., 2011). VPA is a member of the short-chain fatty acid HDAC inhibitor class and is known to be a weak inhibitor of HDAC. However, a case report has been published about the combinatorial usage of valproic acid, cisplatin, and doxorubicin in addition to radiotherapy and surgery to successfully treat a patient with thyroid carcinoma (Noguchi et al., 2008). Currently, VPA is being studied in breast cancer and glial cell tumor in two Phase 2 trials. Furthermore, VPA has shown promising results in neuroblastoma, bladder cancer, thyroid cancer, and breast cancer. VPA induces differentiation and apoptosis through activating Notch signaling in human neuroblastoma cells, reduces proliferation and invasion in bladder cancer cells, increases sensitivity to paclitaxel in thyroid cancer cells, circumvents hypoxia-induced resistance to CDDP-induced apoptosis in neuroblastoma cells, and increases histone H3 acetylation and apoptosis in breast CSCs (Stockhausen et al., 2005; Chen et al., 2006; Catalano et al., 2007; Cipro et al., 2012; Aztopal et al., 2018b). Furthermore, it has been reported that VPA induces apoptosis and sensitizes melanoma cells to cisplatin and etoposide treatment (Valentini et al., 2007). Another FDA-approved HDAC inhibitor, panobinostat, increased PFS and showed a better benefit:risk profile in patients with relapsed or relapsed and refractory multiple myeloma in Phase 3 studies (San-Miguel et al., 2014; Richardson et al., 2016). It has been reported that panobinostat decreased proliferation, increased the expression of tumor suppressor genes in triple negative breast cancer cells, and decreased tumorogenesis in vivo (Tate et al., 2012). Another study showed that panobinostat overcame gefitinib resistance in KRAS-mutant nonsmall cell lung cancer through the downregulation of TAZ (Lee et al., 2017). The 3-deazaneplanocin A (DZNep), a small molecule inhibitor of Ezh2, decreases global H3K27 trimethylation, and its overexpression is associated with aggressiveness of several cancers such as prostate, breast, and bladder, as well as melanoma (Bachmann et al., 2006; Clermont et al., 2017). It has been shown that DZNep causes inhibition of proliferation and migration in mesothelioma (Kemp et al., 2012). Combinatorial therapy with DZNep and nutlin-3 inhibits the interaction between mdm2 and p53-induced apoptosis in HCT116 colon cancer cells through the reduction of bcl-2 expression (Zhou et al., 2018). When DZNep was combined with another HDAC inhibitor SAHA, it induced apoptosis in nonsmall cell lung cancer through the EGFR pathway in vitro and in vivo**(Takashina et al., 2016).Another strategy in epigenetic therapy is targeting BET bromodomain proteins. Bromodomains (BRDs) are “readers” of acetyl marks in histone tails, targeting chromatin-modifying enzymes and other protein machinery to specific sites in the chromatin, thus regulating gene transcription (Pérez-Salvia and Esteller, 2017). JQ1 and IBET762 are 2 well-known selective BET inhibitors (Filippakopoulos et al., 2010). Studies showed that JQ1 blocked binding of the androgen receptor (AR) to the target gene by disrupting BRD4 and AR interaction in castration-resistant prostate cancer (Asangani et al., 2014). JQ1 and IBET762 suppressed proliferation in pancreatic cancer cells as well as inhibited production of nitric oxide and inflammatory cytokines, which are important for the tumor microenvironment in pancreatic cancer cells and immune cells (Leal et al., 2017). PD-L1 expression was inhibited by JQ1 in both immune cells and ovarian tumors in vivo**(Zhu et al. 2016). Da Motta et al. 2017 showed that JQ1 negatively affects tumor response to hypoxia and angiogenesis (da Motta et al., 2017). However, there has been no report regarding the usage of JQ1 in phase trials. On the other hand, there are 6 ongoing phase trials with IBET762 in cancer treatment, especially in solid tumors.All of these studies suggest that when epigenetic drugs are used alone, they have promising results in vitro. On the other hand, combinatorial usage of these drugs with chemotherapy seems more effective in the clinic. It is known that conventional chemotherapeutics target the DNA of rapidly dividing cells. Epidrugs make DNA more targetable for these agents by releasing DNA from the nucleosome and/or allowing the binding of transcription factors to the promoter of specific genes that trigger active gene expression, such as tumor suppressor genes. Furthermore, epidrugs may be more effective in clinical studies due to their immunomodulatory effects, which are necessary for immune cells to target cancer cells.

## 4. Epigenetic status in cancer stem cells

CSCs are a subset of tumor cells which are considered the most important reason for the poor success rate in cancer therapy. Recent studies have revealed that epigenetic alterations in these cells play a key role in the regulation of stemness-related features. It has been shown that differential expression of CD133, a well-known stemness marker, is regulated by epigenetic modifications in many cancer types. The CD133+ population is identified by the promoter hypomethylation in several cancers such as ovarian, glioblastoma, and colorectal when compared to the CD133- population (Tabu et al., 2008; Yi et al., 2008; Baba et al., 2009). It has been demonstrated that TGF-β regulates the CD133 expression through the inhibition of DNMT1 and DNMT3b in the hepatocellular cancer cell line (You et al., 2010). Kagara et al. 2012 reported that hypomethylation of CD44, CD133, and Mushasi-1 promoters is associated with the overexpression of these CSC markers in triple-negative breast tumors (Kagara et al., 2012). DCLK1 (doublecortin-like kinase 1), a CSC marker for intestinal and adenoma cancer, has been recently identified as an epigenetic biomarker of colorectal cancer which is regulated by promoter methylation (Vedeld et al., 2014). Furthermore, CSCs isolated from breast and pancreatic cancer cell lines exhibit higher expression of Ezh2 as compared to the non-CSC populations. Researchers have indicated that RNAi-mediated Ezh2 knockdown leads to decreased frequency of CSCs in these cells, confirming the role of Ezh2 in the maintenance of the CSC phenotype (van Vlerken et al., 2013). CDCP1 (CUB-domain containing protein 1) is a stem cell marker which has been shown to be regulated by epigenetic mechanisms in breast cancer cell lines as well as in clinical findings (Ikeda et al., 2006). Reduction of CDCP1 is associated with tumor invasiveness and poor prognosis in esophageal cancer (Sawada et al., 2014). These results suggest that dynamism in DNA methylation as well as histone modifications contribute to tumor heterogeneity through the transition between the active and repressive state of stemness-related genes and represent a potential target for epigenetic therapy.

### 4.1. Targeting DNA modifications in CSC therapy

It has been mentioned above that DNMT inhibitors are highly toxic as cytotoxic agents, but in low doses they have great efficacy in maintaining reduced DNA methylation and reexpression of silenced genes in leukemic and epithelial tumor cells (Tsai et al., 2012). It has been predicted that they may reduce tumor formation in cancer patients and target CSC populations within the tumor. Liu et al. 2015 demonstrated that regulated inhibition of DNMT1 was able to reduce the proliferation and tumorigenic ability of lung CSCs (Liu et al., 2015). It has been reported that decitabine-treated prostate cancer CSCs exhibited diminished expression in stemness genes OCT4 and Nanog, leading to a general decrease in tumor growth (Tian et al., 2012). SGI-110, a novel DNMT inhibitor, has been reported to reprogram ovarian CSCs to a more differentiated state (Wang et al., 2014). It has been reported that EPG induced apoptosis in lung CSCs through hsa-mir-485-5p upregulation and RXRα downregulation, which are CSC markers (Jiang et al., 2018). Chen et al. 2017 reported that EPG showed an antiproliferative effect, induced apoptosis, and reduced stem cell properties by inhibiting the Wnt pathway in colorectal CSCs (Chen et al., 2017). It has been stated that EPG inhibits STAT3 activation; hence, STAT3 target genes related to stemness characterization in breast CSCs are downregulated (Chung et al., 2015). Toden et al. 2016 demonstrated that EPG induced apoptosis, caused cell cycle arrest, and diminished CSC formation in colorectal CSCs (Toden et al., 2016).

### 4.2. Targeting histone modifications in CSC therapy

Therapy resistance is an important issue to overcome in cancer treatment. This resistance is mostly associated with CSCs and epigenetic regulation (Sharma et al., 2010). SAHA and romidepsin are HDAC inhibitors that have been approved for treatment of cutaneous T-cell lymphoma. Both drugs were found to exhibit durable response and efficacy in patients with cutaneous T-cell lymphoma (Olsen et al., 2007; Piekarz et al., 2009; Whittaker et al., 2010; Thaler et al., 2012). It has been reported in our previous study that VPA negatively regulated self-renewal capacity and induced caspase-independent apoptosis in breast CSCs (Aztopal et al., 2018b). Higher activity of Ezh2 has been reported in different cancers such as breast, lung, and prostate, as well as hematological malignancies, and is associated with poor disease prognosis (Kleer et al., 2003; Weikert et al., 2005; Simon and Lange, 2008; Li et al., 2013; Wang et al., 2016). Further studies have also suggested the role of Ezh2 deregulation in tumor progression, metastasis, and maintenance of CSCs’ self-renewal properties (Bracken et al., 2006; Suva et al., 2009; Shi et al., 2013). Furthermore, it has been reported that inhibition of Ezh2 by DZNep was able to reduce self-renewal and tumor-initiating features of glioblastoma multiforme CSCs in vivo through the transcriptional regulation of oncogene MYC (Suva et al., 2009).

### 4.3. Combination therapy with epigenetic modulators in CSC therapy

It has been highlighted in several recent studies that the use of epigenetic drugs in combination with conventional chemotherapy agents resensitizes resistant CSCs to drug treatment, or primes cancer cells for subsequent therapies (Easwaran et al., 2014; Juo et al., 2015). For instance, low doses of SGI-110 (DNMT inhibitor) was established to impel ovarian CSCs toward a more differentiated phenotype and sensitize them to platinum treatment (Wang et al., 2014). Li et al. 2015 reported that encapsulating decitabine and doxorubicin in nanoparticles helped to better target breast CSCs and inhibit tumor growth (Li et al., 2015). In a recent study, coinhibition of Wnt, HDAC, and Erα (estrogen receptor alpha) has been shown to effectively suppress the breast CSC population. When tamoxifen was added to this combination, it was reported that the CSC population was successfully suppressed at lower doses (Sulaiman et al., 2016). Chikamatsu et al. 2013 showed that HDAC inhibition in head and neck cancer exhibited a synergistic effect with chemotherapeutic agents, suppressed stem cell character and EMT, stopped the cell cycle, and induced apoptosis (Chikamatsu et al. 2013). It has been reported that EPG sensitized colorectal cancer cells to 5-FU (Toden et al., 2016). Recently, our research group demonstrated that the combination of VPA with niclosamide (Wnt/β-catenin signaling inhibitor) increased histone H3 acetylation and induced extrinsic apoptosis in breast CSCs in vitro (Aztopal et al., 2018a). 

## 5. Emerging role of epitranscriptome in cancer therapy

Epitranscriptome refers to the posttranscriptional regulation of gene expression via chemical RNA modifications that do not change the RNA sequence (Saletore et al., 2012). RNA modifications have begun to be appreciated as emerging players in the regulation of various biological processes, such as the maintenance and differentiation of embryonic stem cells, modulation of circadian rhythm, and stress response, as well as tumorigenesis (Fustin et al., 2013; Geula et al., 2015; Popis et al., 2016; Zhang et al., 2017). Although more than 150 RNA modifications are defined, the physiological significance of only a few is known, especially in tumorigenesis. Furthermore, it has been shown that these modifications are dynamic and reversible like epigenetic alterations, implying that their biological significance in cancer therapy is comparable with DNA and histone modifications (Zhao et al., 2017). Hence, RNA epigenetics has become an attractive research area due to the discovery of their targetable features in cancer therapy.N6-methyladenosine (m6A) is the most common modification in eukaryotic mRNAs and is well-studied in cancer research (Desrosiers et al., 1974). Recent accumulating evidence suggests that it has been a key player in cancer-related hallmarks including cancer stemness, EMT (epithelial-mesenchymal transition), and drug resistance. Zhang et al. 2016 reported that activation of ALKBH5 that demethylates m6A promotes the stemness of breast CSCs (Zhang et al., 2016). Similarly, it has been demonstrated that knockdown of METTL3 and METTL14 which catalyzes the formation of m6A modification triggers the growth and self-renewal of glioblastoma stem cells (Cui et al., 2017). Li et al. 2017 showed that upregulation of METTL3 inhibits invasion and migration capacity as well as EMT in renal cell carcinoma in vitro and suppresses tumor growth in vivo (Li et al., 2017). Moreover, it has been stated that overexpression of YTHDF1 (m6A binding protein, reader) in colorectal cancer is associated with poorer overall survival, and that knockdown of YTHDF1 in vitro sensitizes cells to anticancer drugs (Nishizawa et al., 2018). Overall, these results suggest that manipulation of m6A levels modulates aggressive tumor character and drug response, implying its eligibility as a therapeutic target in drug discovery.

## Conclusion

In conclusion, as given above, the potential of epigenetic drugs in terms of their activity against solid tumors is huge. Although there have been some failures of epigenetic therapy on cancer, it is still of immense importance and shows great promise for better outcomes for cancer patients. Possibly the future discovery of various small molecules which target the epigenetic regulators might result in novel therapy modalities with more specific actions. As given in detail above, the combination of epigenetic agents with classical chemotherapy agents and/or immunotherapy is a developing and especially promising option for cancer patients which will surely make a great impact on cancer treatment in the near future.
